# Automatic Sorting System for Rigid Piezoelectric Transducer Wafers Used in Displacement Adjustment

**DOI:** 10.3390/mi11100915

**Published:** 2020-09-30

**Authors:** Tongqun Ren, Xin Li, Xiaodong Wang, Zheng Xu, Yugang Liu, Jin Yang, Jiang Guo

**Affiliations:** 1 Key Laboratory for Precision & Non-traditional Machining of the Ministry of Education, Dalian University of Technology, Dalian 116024, China; ren_tq@dlut.edu.cn (T.R.); xuzheng@dlut.edu.cn (Z.X.); guojiang@dlut.edu.cn (J.G.); 2 Key Laboratory for Micro/Nano Technology and System of Liaoning Province, Dalian University of Technology, Dalian 116024, China; duter_xin@163.com; 3 AVIC Xi’an Flight Automatic Control Research Institute, Xi’an 710076, China; boylyglyg@163.com (Y.L.); 5813091@163.com (J.Y.)

**Keywords:** piezoelectric transducer wafer, resonant cavity length, piezoelectric coefficient, deformation measurement, sorting system

## Abstract

Piezoelectric transducer wafers are usually used in pairs to adjust the resonant cavity length of the ring laser gyro. In practice, the paired wafers are required to have similar piezoelectric charge coefficient d_31_. To handle the pairing operation in-batch, an automatic sorting system was developed on the basis of deformation measurement, which adopted a frame of a Cartesian-coordinate robot. The wafers were self-aligned in the vertical direction, and a vacuum holder was used to pick up, transfer, and then place them on thee testing desk one by one. The excitation voltage was loaded on the wafer by a specifically designed electrode, and the resulting micro deformation was measured by dual opposite inductive micrometers using the relative measurement principle. This particular electrode has the function of attitude self-adjustment and vacuum adsorption, which is conducive to loading the voltage reliably and protecting the wafer from undesired damage. Finally, the wafers were transported to different stock bins based on the measuring results. This system is suited to handle a mass of wafers by continuous processing on site for its high reliability and measurement consistency. The measurement accuracy, validated by laser interferometry, was better than 0.5 μm and the repeatability was superior to 0.1 μm.

## 1. Introduction

As the key device for strap-down inertial navigation system, laser gyro is widely used in aerospace field [1,2,3,4]. The optical length of the ring resonant cavity is the product of the medium refractive index in cavity and resonant cavity length. Its minor response leads to the deviation of the working central frequency and then causes a fatal impact on the working stability of the laser gyro. One method to stabilize the working central frequency is to adjust the medium refractive index in the cavity such as the temperature compensation method [5,6,7,8]. Another frequency stabilization method is to adjust the length of the resonant cavity directly, where piezoelectric transducer wafers are usually used in a pair to drive the mobilizable mirror in the ring resonant cavity [9,10,11,12,13]. The wafers should be paired according to their deformation mainly caused by the reverse piezoelectric effect. Therefore, sorting and pairing should be performed before the use of piezoelectric transducer wafers.

In industry, the pairing of piezoelectric transducer wafers is normally based on manual measurement. The efficiency of manual measurement is low, and the results are greatly affected by the operation of workers. It is difficult to guarantee the measurement quality, which seriously limits the industrialization of subsequent assembly for laser gyro. To meet the requirement of production capacity, an automatic sorting system that deals with measuring and pairing piezoelectric transducer wafers in-batch is extremely necessary.

Optical and electrical methods are mainly applied to deal with micron-sized deformation measurements of piezoelectric transducer wafers. For instance, laser interferometry is widely used as it processes high resolutions of 10^−14^ m [14,15,16,17]. However, an interferometry system is usually sensitive to the working conditions. The operators are also required to possess high skillful technical talent. Moreover, the system is always ponderous and expensive due to complex optical components. Therefore, laser interferometry is mainly used in the laboratory for small batch and high precision measurement tasks. The digital image correlation (DIC) method is widely used in the field of micro-operation/microfluidics systems [18,19] and is also applied to measure piezoelectric strain coefficients [20]. For the DIC method, the test surface should have a unique pattern for subset tracking to identify the subsets from each other. When the natural surface texture cannot provide sufficient identification marks, the unique pattern must be created manually for each wafer, which is not convenient in batch measurement. The electrical methods mainly include the capacitive method [21,22,23] and inductive method [24,25]. In the capacitive method, a parallel plate capacitive sensor is always used, and one electrode plate is in a mechanical serial connection with one end of the measured wafer. In inductive method, a non-contact inductive proximity sensor is always used, and similarly, a metal plate must be affixed to the end of the measured wafer to make the sensing coil work. Just like creating artificial marks in the DIC method, the connection or affixation operations need to be performed for each wafer, which is inconvenient for batch measurement.

For a millimeter size hard piezoelectric transducer wafer, its deformation measurement comes to a common problem of micro displacement measurement, and contact measurement can also be competent. Comparatively speaking, the inductive micrometer is the preferred sensor because of its simple operation, stable performance, high reliability, and low requirements for working conditions [26,27]. Moreover, the inductive micrometer is easy for system integration to cooperate with automated production.

In this work, in order to deal with the pairing of piezoelectric transducer wafers, an automatic sorting system based on in-site batch micro-deformation measurement was developed. A Cartesian-coordinate robot frame was adopted as the main body, and relative measurement principle is employed by using dual opposite inductive micrometers.

## 2. Structural Systematic Design

### 2.1. Overall Design

**Figure 1:**
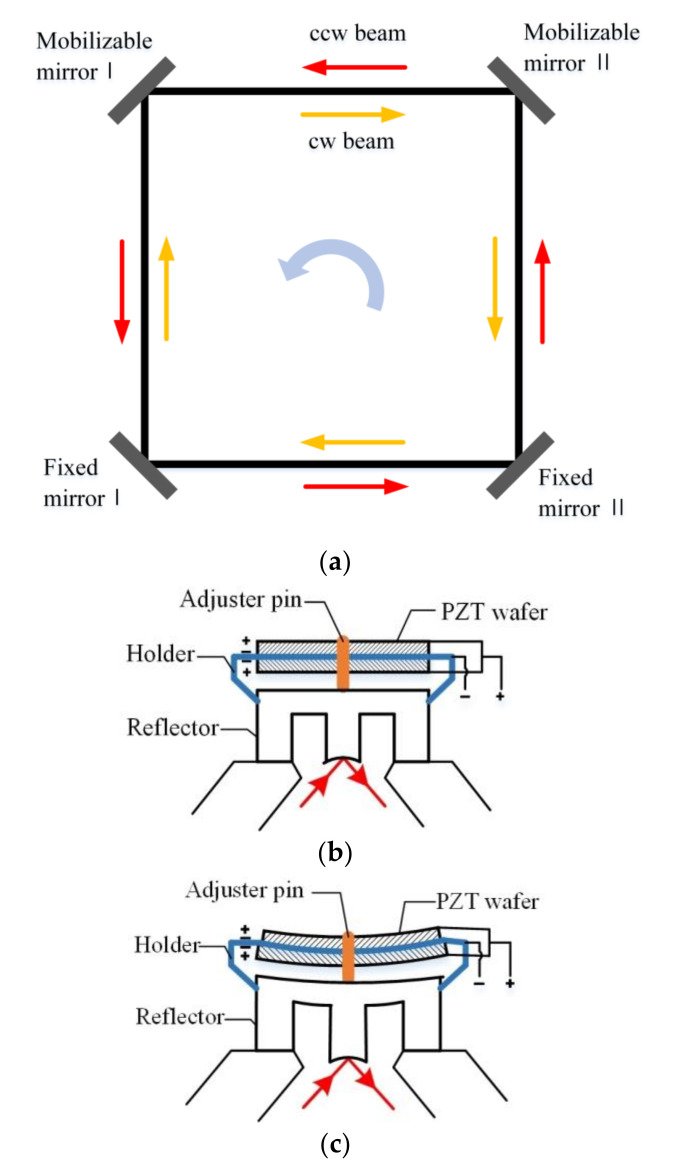
The basic setup of a square ring laser gyro. (**a**) Overall perspective of square ring laser gyro. (**b**) Detail of mobilizable mirror. (**c**) Adjustment principle.

The stability of the working central frequency of the laser gyro is influenced by the variation in the optical length of the ring resonant cavity. When piezoelectric transducer wafers are used for resonant cavity length adjustment, the basic setup of a square ring laser gyro can be simplified, as shown in Figure 1.

Two wafers are stacked together between an intermediate holder. When the excitation voltage is applied, the two wafers deform in the opposite direction. In this case, the holder bends and drives the adjuster pin to push or pull the reflector. Thus, the length of resonant cavity is adjusted, as shown in Figure 1b. In practical application, the paired wafers are required to have a similar piezoelectric property. Therefore, the sorting of the piezoelectric transducer wafer must be established on the deformation measurement.

**Figure 2:**
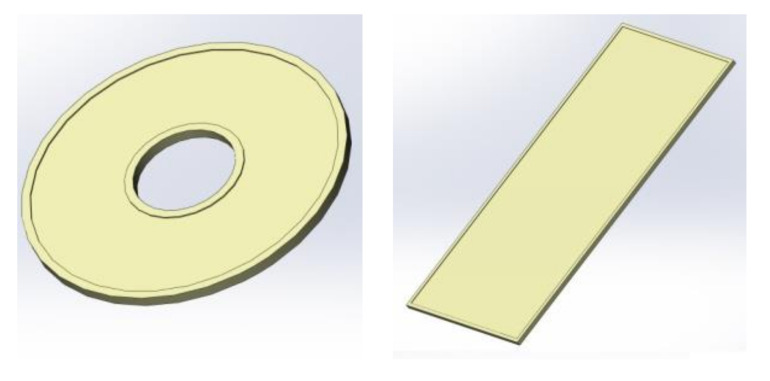
The most commonly used piezoelectric transducer wafers.

The most commonly used piezoelectric transducer wafers for laser gyro have annular or rectangular shapes, as shown in Figure 2.

Generally, the basic sorting process can be set as the following continuous operations:

1. Pick up the wafer to be tested from a feeding platform.
2. Transfer the wafer and place it on a measurement platform.
3. Apply the excitation voltage and then measure the corresponding deformation.
4. Unload the excitation voltage and remove the wafer to a certain recovering platform, according to the measurement results.

**Figure 3:**
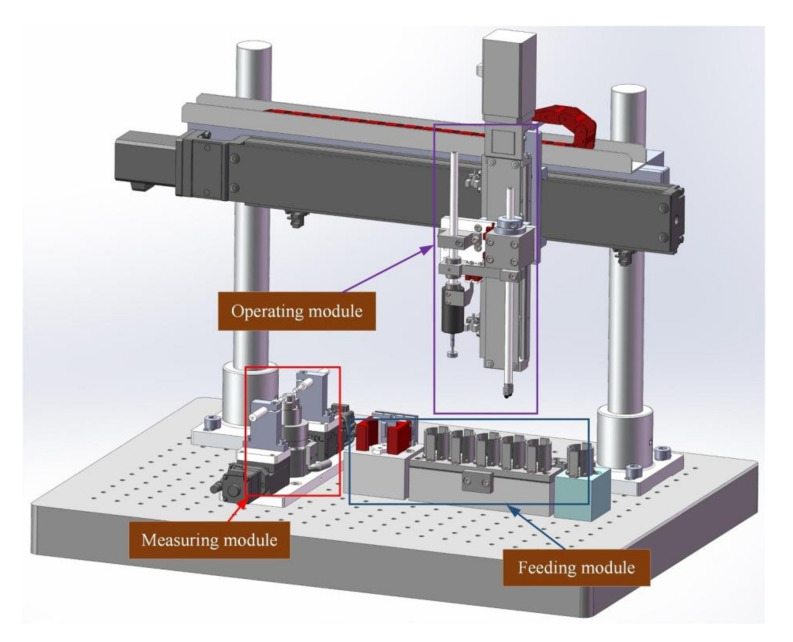
The overall design scheme of this system.

According to the user requirements, the design objectives of this system are as follows. The measurement accuracy in full scale was no more than 0.5 µm and the repeat accuracy was superior to 0.1 µm. To accomplish the above operations, three main functional modules were designed and integrated to form the sorting system. The feeding platform and recovering platform were grouped together, which is collectively referred to as the feeding module. A pick-up arm and electrode were integrated to a Cartesian-coordinate robot frame to construct the operating module. The operating module realizes the operations of picking up, transferring, and placing the wafer as well as loading/unloading the excitation voltage. The measuring module was composed of a testing desk and dual opposite inductive micrometers carried by a respective linear guide rail, which realized the measurement of micron-sized deformation. Note that the target system is used for measuring and pairing piezoelectric transducer wafers in-batch. The accuracy of measurement and process safety in use must be ensured at the same time. Therefore, the main body of this system was installed on an optical platform, and multiple rubber vibration isolation cushions were set under the platform to achieve good stability. Furthermore, a special electrode was designed carefully and reliable hardware protection was also adopted in corresponding modules, which will be expanded in the next section. To sum up, the overall design scheme of the sorting system is shown in Figure 3.

### 2.2. Functional Modules

#### 2.2.1. Feeding Module

**Figure 4:**
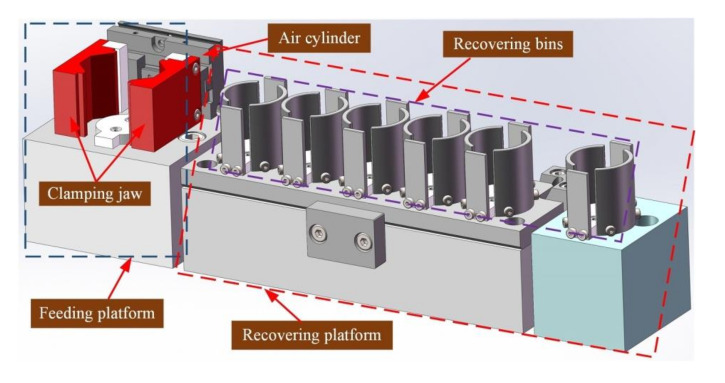
Structure of the loading/blanking module.

Figure 4 demonstrates the detail of the feeding module. An air cylinder carrying centering clamping jaws were mounted on a supporting seat to form the feeding platform. A total of six stock bins were mounted on other supporting seats to form the recovering platform. One of the stock bins was separated from the others for an unqualified wafer. The stock bins were constructed by a combination of arc baffles and panel baffles to meet both annular and rectangular wafers. 

Before measurement, the wafers were freely stacked on the feeding platform. Then, the cylinder drives the clamping jaw to tighten inward, which completes the central alignment of the wafers. Therefore, only vertical motion is required to be controlled during the pick up operation. The clamping jaw was designed as a V-shape with small flat surface at the top for adapting to different shapes of wafers. After measurement, the wafers were sorted based on the measuring results.

#### 2.2.2. Operating Module

**Figure 5:**
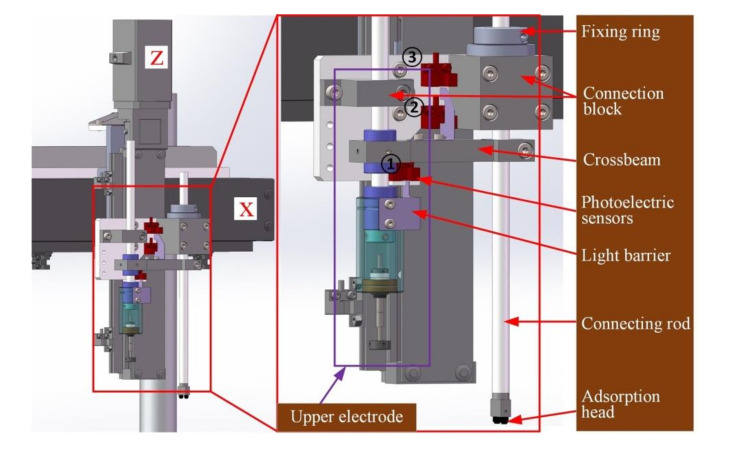
Structure of the operating module.

As shown in Figure 5, a Cartesian-coordinate robot frame, actually a planer type structure consisting of an x-axis and z-axis, was used for the operating module. The x-axis was used for horizontal motion to transfer the wafer, and the z-axis was used for vertical motion to pick up, place the wafer, and apply voltage to it. Since the wafer is hard and brittle, and multiple wafers are stacked during the measurement, it is inconvenient to pick up a wafer by mechanical clamping. Therefore, a pick-up arm based on a vac-sorb device was designed.

The pick-up arm was mounted on z-axis through a connection block in which a linear bearing was embedded. A hollow connecting rod passed through the linear bearing so that it had a vertical degree of freedom of motion. A fixed ring was arranged at the top of the connecting rod to avoid it falling off due to gravity. An adsorption head was installed at the bottom of the connecting rod. Since the annular wafer had an inner hole, double rubber vacuum chucks were adopted to ensure stable picking operation. The connecting rod is connected to a vacuum generator through a gas-guide tube.

**Figure 6:**
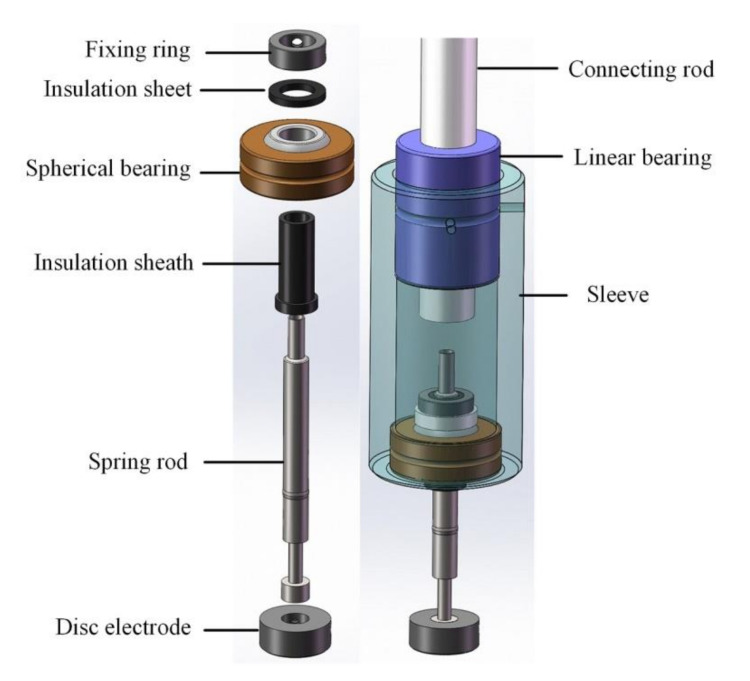
Structure of the upper electrode.

The upper electrode employs split-type design, as shown in Figure 6. A hollow connecting rod was mounted on the z-axis through another connection block, and was also connected with the sleeve through an embedded linear bearing at the same time. A spherical bearing was mounted at the bottom of the sleeve. The stretchable electrode, combination of a spring rod and a disc electrode, was connected to the spherical bearing by a fixing ring and insulation sheet/sheath. Similarly, the fixing ring prevented the electrode from falling off. In particular, the utilization of linear bearing and spherical bearing made the upper electrode achieve both vertical motion freedom and ability of attitude self-adjustment. This well-designed structure made the working surface of the disc electrode contact the wafer completely with constant contact force during the excitation voltage loading operation.

The pick-up arm was parallel to the upper electrode and rigidly connected by a crossbeam, which was physically separated from the z-axis. To realize the limit and hardware protection of operating module, the z-axis was equipped with three additional photoelectric sensors. In the pick-up operation, the z-axis descends to make the adsorption head touch the wafer. Under the joint action of contact force and linear bearing, the pick-up arm moves upward together with the crossbeam relative to the z-axis. When the correct preset position is reached, the light barrier mounted on the crossbeam triggers photoelectric sensor 2 (see Figure 4 for details) to generate a stop signal for the z-axis. If the z-axis fails to respond correctly due to unexpected circumstances, it continues to descend and the light barrier continues to move up to trigger photoelectric sensor 1. Right now, an emergency stop signal is generated. This signal strictly stops all the guide rails simultaneously through interruption control. This protection strategy avoids damage on the system hardware and the wafer caused by excessive motion of the z-axis.

#### 2.2.3. Measuring Module

**Figure 7:**
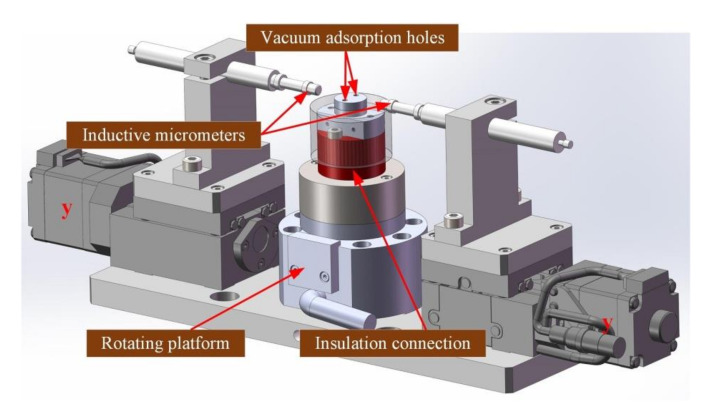
Structure of the measuring module.

The measuring module realized the loading of excitation voltage and micro-deformation measurement of the piezoelectric transducer wafer. It was composed of a testing desk and dual opposite inductive micrometers carried by the respective linear guide, as shown in Figure 7.

The testing desk was placed in the middle of the measuring module, which is actually an integration of an electric rotating platform and a lower electrode. An insulation connection was also used to guarantee the electric isolation. The electric rotating platform was used to measure the displacement of the wafer in different directions if required (here, the measurement was performed in one direction). Two y-guide rails were mounted on both sides of the testing desk, each carrying an inductive micrometer. During the measurement operation, the pick-up arm placed the measured wafer on the lower electrode and then moved away. Subsequently, the upper electrode moved down to press on the wafer. The upper electrode was also equipped with a light barrier on its sleeve. Thus, the position control and hardware protection was realized similar to that of the pick-up arm. The y-guide rails drive two inductive micrometers to move in the opposite directions until both micrometers touch the wafer. Finally, the excitation voltage was loaded on the wafer through cooperation of the upper and lower electrodes. Based on the relative measurement principle, the deformation can be calculated by the readings of two inductive micrometers before and after voltage loading.

The piezoelectric transducer wafer was made of ferroelectric piezoelectric dielectric materials. Under the action of the electric field, it deformed due to characteristics of the electrostrictive effect, inverse piezoelectric effect, and ferroelectric effect. Generally, the electrostrictive effect is extremely weak compared with other effects. As a result, the deformation caused by the electrostrictive effect is lower by 1–2 magnitudes than that caused by the other two effects, which can be ignored. The deformation caused by the inverse piezoelectric effect is linear, and the deformation caused by the ferroelectric effect is nonlinear, which is related to the angle between the direction of spontaneous polarization and the electric field, and is mainly manifested as hysteresis nonlinear. Therefore, the deformation of the measured piezoelectric transducer wafer is nonlinear [28]. In this work, our aim was to pair the piezoelectric transducer wafers that had close deformations under action of the same electric field, so we did not care whether the deformation was linear or not.

To avoid lateral sliding of the wafer during impaction operation of the upper electrode and rotation of rotating platform, the lower electrode was also connected to a vacuum adsorption device. The wafer was adsorbed on the lower electrode, until the impaction operation was completed.

All guide rails used in the above functional modules were from the series XCVLC××× from MISUMI (China). The micrometer used in this system was a GTL222-A from TESA SA (Lausanne, Switzerland). The major parameters of GTL222-A are summarized in Table 1.

In fact, our system has the characteristics of micro-operation, and even the design idea and main structure of our system can be transferred to a micro-operation just like the experimental setup in [18,19] after an appropriate improvement.

#### 2.2.4. Control System

**Figure 8:**
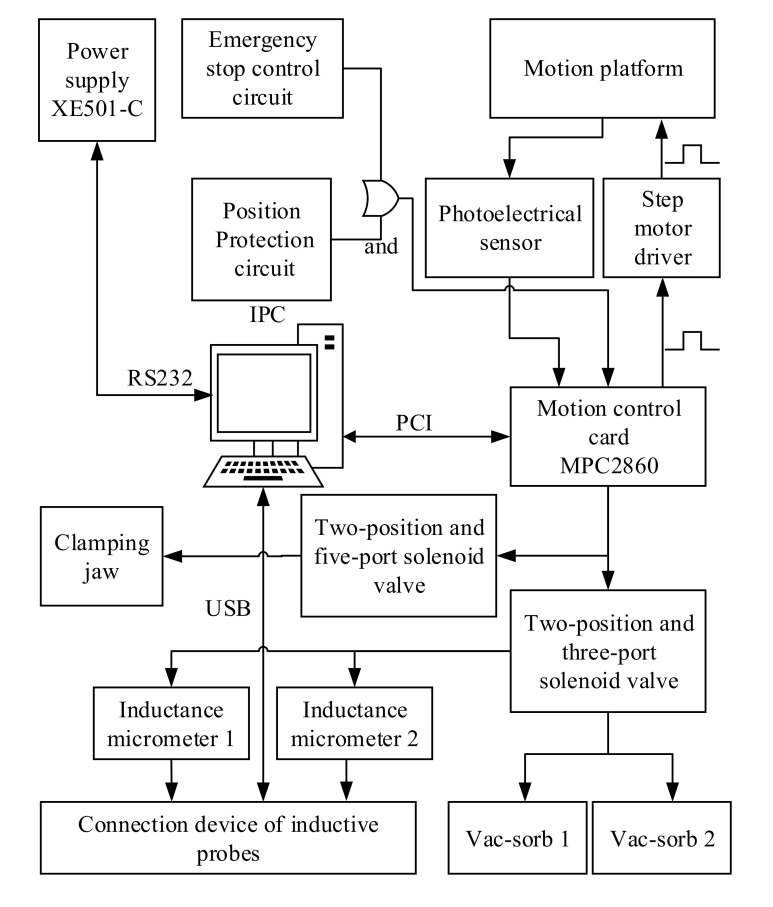
Schematic diagram of the circuit control system.

The electronic control system is briefly illustrated in Figure 8 and deals with the motion control of multiple guide rails, data acquisition of inductive micrometers, and photoelectric sensors. The motion control is fulfilled by the method of “IPC + Motion control card (MPC08E from LEETRO Automation Co., Ltd., Chengdu, China) + Step motor driver (MD5-HD14 from Autonics, Korea)”. The motion control card also monitors the stop signal from the photoelectric sensors. An emergency stop button was set to deal with emergency situations such as hardware conflict. When it is pressed, an emergency stop signal is generated, just like that from photoelectric sensor 1. The motion control card detects this signal by its external interrupt port, and then stops outputting all motion driving signals.

**Figure 9:**
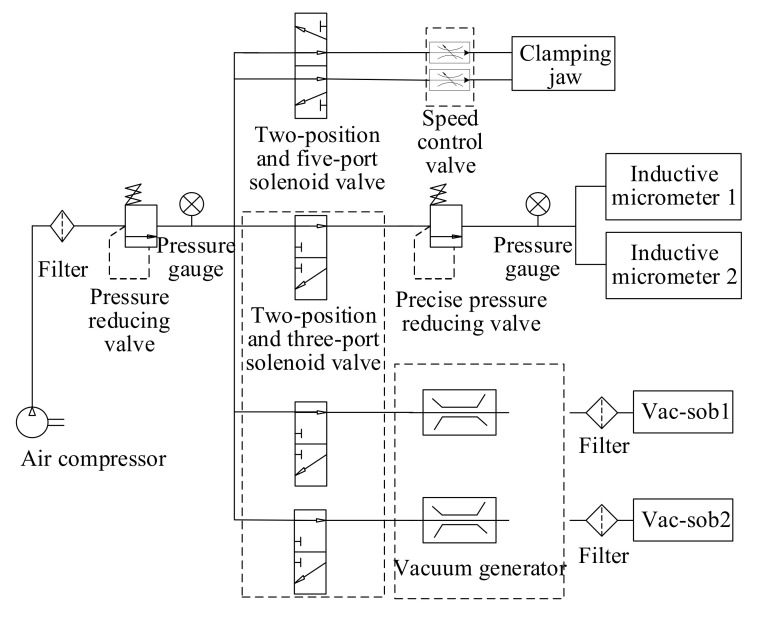
Schematic diagram of the pneumatic control system.

This equipment involves three types of pneumatic components (i.e., two vacuum holders, inductive micrometers, and a bidirectional cylinder in feeding platform). Therefore, there are three branches in the pneumatic control system, as shown in Figure 9. Since the inductive micrometers need relative low and stable air pressure to ensure their normal work state, a secondary precision pressure reducing valve was set in the corresponding control branch.

## 3. Experiments and Discussion

**Figure 10:**
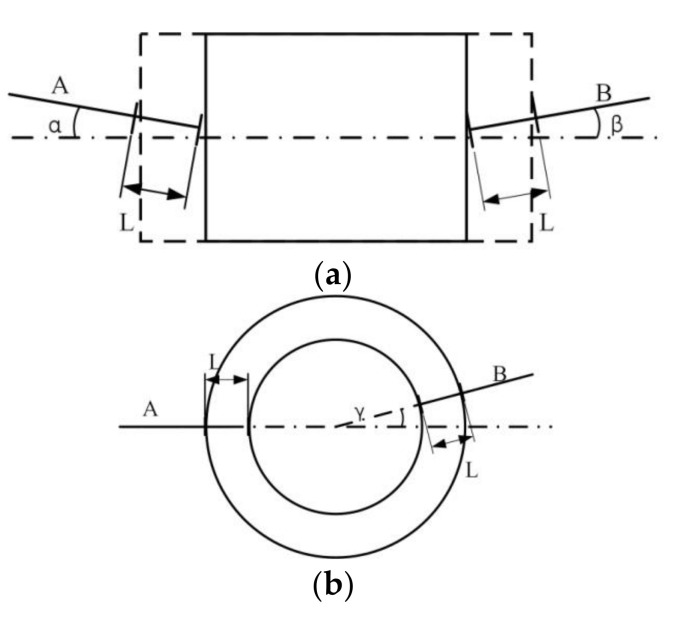
Error analysis in the horizontal plane. (**a**) Analytical model for rectangular wafer. (**b**) Analytical model for annular wafer.

The relative measurement principle determines that the accuracy of the moving platform in all modules has no effect on the final measurement results. In fact, one of the error sources is the installation orientation of two inductance micrometers. In general, it is assumed that the measured wafer is placed in the horizontal plane. For a rectangular wafer, the installation direction of inductance micrometers A and B deviates from the deformation direction by angle *α* and *β*, respectively, and the measured value for each inductance micrometer is L, as shown in Figure 10a. Thus, the measuring error Δ*d*_install_ caused by the installation orientation can be calculated as:

(1) $$\Delta d_{\mathrm{install}}=L\times \left(2-\cos\alpha -\cos\beta \right)$$

Since the installation angle *α* and *β* are very small, which can be controlled within 1° in practice. It can be guaranteed that Δ*d*_install_ < 3.04 × 10^−4^ × L, where L is no more than 6 µm. The measuring error caused by the installation angle in the vertical plane has the same order of magnitude as Δ*d*_install_. They can both be ignored. For an annular wafer, the deformation is along the radial direction, as shown in Figure 10b. Therefore, there is no measuring error in horizontal plane theoretically. Such measuring errors in the vertical plane can be also ignored.

In this system, the excitation voltage is required to be stable and programmable with high control precision. Here, a dedicated power supply (XE501-C) for piezoelectric transducer was employed, whose control precision was ±0.5 V in full scale. The key parameters of XE501-C are listed in Table 2. 

Usually, the inverse piezoelectric effect of the piezoelectric transducer wafer (i.e., deformation-voltage curve) is linear. According to the user, the piezoelectric charge coefficient d_31_ of the measured wafer was less than 0.02 µm/V. According to Table 2, the deformation of the measured wafer was less than 0.02 µm/V × 300 V = 6 μm. The voltage fluctuation ΔU at full-scale output was about 0.1% × 300 + 0.5 + 0.005 ≈ 0.8 V. Therefore, the error Δ*d*_U_ caused by the power input for inverse piezoelectric effect was no more than 0.02 µm/V × 0.8 V = 0.016 μm << 0.5 µm.

For the ferroelectric effect, the deformation *D_f_* can be formulated by:

(2) $$D_{f}=\sum l_{i}\left(\cos\alpha_{ie}-\cos\alpha_{io}\right)$$

where *l_i_* is the axial length of spontaneous polarization of the *i*th electric domain; *α_ie_* is the angle between the direction of spontaneous polarization and the electric field of pre-polarization; and *α_io_* is the angle between the direction of spontaneous polarization and the electric field of action. The larger the voltage, the closer the spontaneous polarization direction to the field strength direction (i.e., the bigger (*α_ie_* − *α_io_*) is). It is hard to quantify the error caused by the power input for the ferroelectric effect. Generally, the deformation caused by the hysteresis nonlinearity shall not exceed 15% of the total deformation. By employing the driving method of hysteresis resistance, the hysteresis nonlinearity can be reduced by 90 percent [29,30]. Thus, the error caused by hysteresis nonlinearity is much less than 0.15 × 0.1 × 6 μm = 0.09 μm.

According to Table 1, the accuracy of the micrometer GTL222-A was 0.2 + 2.4 L^2^, where 0.2 is the basic error term and L is the real stroke (measured deformation) of the probe (mm). In this work, the real deformation L was 0~6 µm. Therefore, the error term of 2.4 L^2^ can be calculated as 2.4 × (0~0.006)^2^ µm, which can be ignored. For the basic error term, 0.2 was actually the maximum value in the total measurement range of ±1.5 mm. During measurement, the probe of the micrometer is continuously compressed by micron-sized L in the same single direction. In this case, the value of the basic error in two consecutive measurements will not jump distinctly. Therefore, for relative measurement, the basic errors of two inductive micrometers can be considered to mutually offset each other to some extent. From a cautious point of view, the error Δ*d*_micrometer_ caused by inductive micrometers was less than 0.2 μm due to micron-sized L.

Based on the above analysis, the measurement model of the deformation of the piezoelectric transducer wafer can be established as follows:

(3) $$D=d_{31}\times \left(U+\Delta U\right)+\sum l_{i}\left(\cos\alpha_{ie}-\cos\alpha_{io}\right)+\Delta d_{\mathrm{micrometer}}+\Delta d_{\mathrm{install}}$$

where *U* is the expected excitation voltage and *D* is the measured value of total deformation. Then, the composite error of *D* can be calculated by:

(4) $$\Delta D=\pm t\sqrt{\sum_{i=1}^{q}{\left(\frac{a_{i}\Delta d_{i}}{t_{i}}\right)}^{2}+2\sum_{1<i<j}^{q}\rho_{ij}a_{i}a_{j}\frac{\Delta d_{i}}{t_{i}}\frac{\Delta d_{j}}{t_{j}}}$$

where *q* is the number of error terms; *a_i_* is the propagation coefficient of single error; *t_i_* is the confidence coefficient of single limit error; and *t* is the confidence coefficient of the total limit error. As each single error obeyed the normal distribution in this work, the confidence coefficients of single limit error and the total limit error were the same. That is, *t*_1_ = *t*_2_ = ··· = *t_q_* = *t*. Moreover, each single error is independent, which leads to *ρ_ij_* = 0. At this time, the composite of the limit error can be calculated as:

(5) $$\Delta D=\pm \sqrt{\sum_{i=1}^{q}{\left(a_{i}d_{i}\right)}^{2}}$$

Suppose the installation angle *α* is equal to *β*, *a_i_* can be given by:

(6) $$\left\{\begin{array}{l} a_{1}=\partial D/\partial U=d_{31}+\partial \left[\sum l_{i}\left(\cos\alpha_{ie}-\cos\alpha_{io}\right)\right]/\partial U\\ a_{2}=\partial D/\partial \Delta d_{\mathrm{micrometer}}=1\\ a_{3}=\partial D/\Delta d_{\mathrm{install}}=1 \end{array}\right.$$

The propagation coefficient *a*_1_ is related to the manufacturing process and measurement condition, especially U. Therefore, *a*_1_ is hard to quantify. It is reasonable to give the error range by adding up the extreme value of each single error, although it will be greater than the actual error. Therefore, the error of this system was no more than 3.04 × 10^−4^ × 6 μm + 0.016 μm + 0.2 μm + 0.09 μm ≈ 0.3 μm.

**Figure 11:**
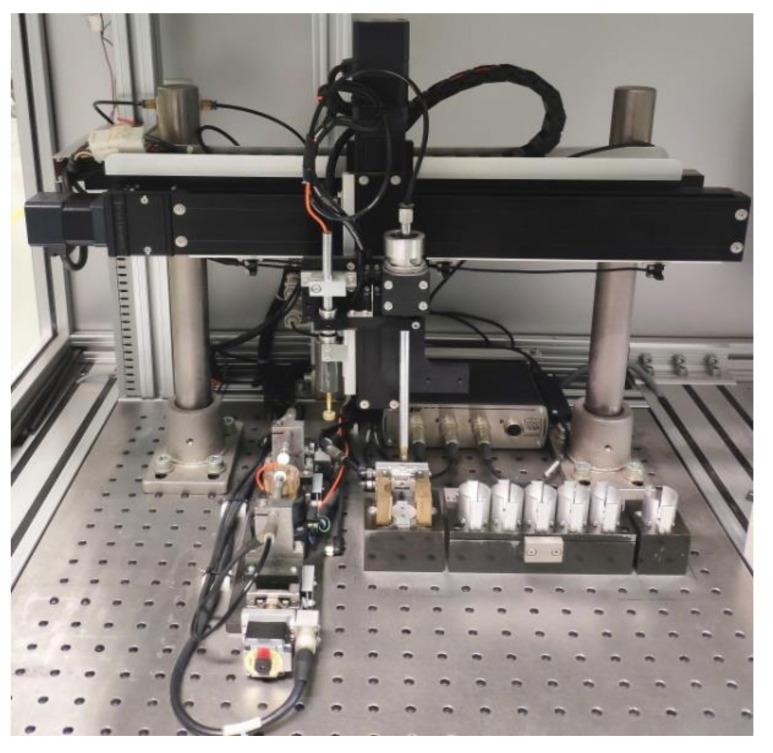
The physical sorting system for the piezoelectric transducer wafer.

The actual system is illustrated in Figure 11. Aside from the theoretical analysis, a series of experiments for actual piezoelectric transducer wafers were carried out to verify its feasibility. So far, it has been operating in the user unit for nearly two years.

**Figure 12:**
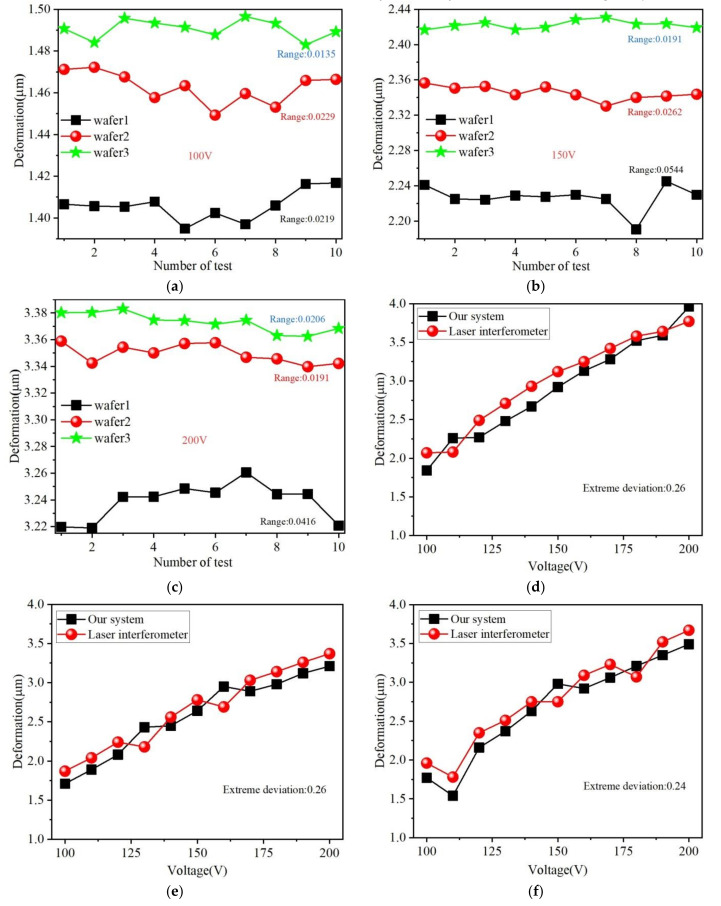
Measurement results of the three arbitrary annular/rectangular wafers at specific voltages of (**a**) 100 V for annular test sample 1, (**b**) 150 V for annular test sample 2, (**c**) 200 V for annular test sample 1, (**d**) 100 V~200 V for rectangular test sample 1, (**e**) 100 V~200 V for rectangular test sample 2, and (**f**) 100 V~200 V for rectangular test sample 3.

First, three arbitrary annular wafers were measured 10 times at the specific voltages of 100 V, 150 V, and 200 V, respectively. The measurement was performed continuously in a clean shop with constant temperature and humidity. Moreover, the voltage was also loaded in the same way. The measuring results are illustrated in Figure 12a–c, where the maximal range of measuring result for the same wafer was about 0.05 µm < 0.1 µm. This demonstrates that the repeat accuracy of our system met the design objective. Of course, the repeatability (experimental standard deviation) *s*(*x_k_*) and uncertainty (Type A standard uncertainty) u(*x_k_*) of each measurement was calculated by the Bessel formula as follows:

(7) $$s\left(x_{k}\right)=\sqrt{\frac{1}{n-1}\sum_{i=1}^{n}{\left(x_{i}-\bar{x}\right)}^{2}},u\left(x_{k}\right)=s\left(x_{k}\right)/\sqrt{n}$$

Thus, the maximum value of *s*(*x_k_*) occurred when wafer 1 was tested at 150 V, which was 0.015 µm, and the corresponding maximum value of uncertainty *u*(*x*) was no more than 0.16%.

Second, three arbitrary rectangular wafers were measured 10 times at different voltages from 100~200 V by our system and laser interferometer (Aglient 5530), respectively. The measurement experiment was performed continuously in the same clean shop and the delay time of two kinds of measurements did not exceed 10 min. The measuring results are illustrated in Figure 12d–f, where the extreme deviation between the results of our system and laser interferometer was 0.26 µm < 0.5 µm. This demonstrates that the measurement accuracy of our system meets the design objective.

**Figure 13:**
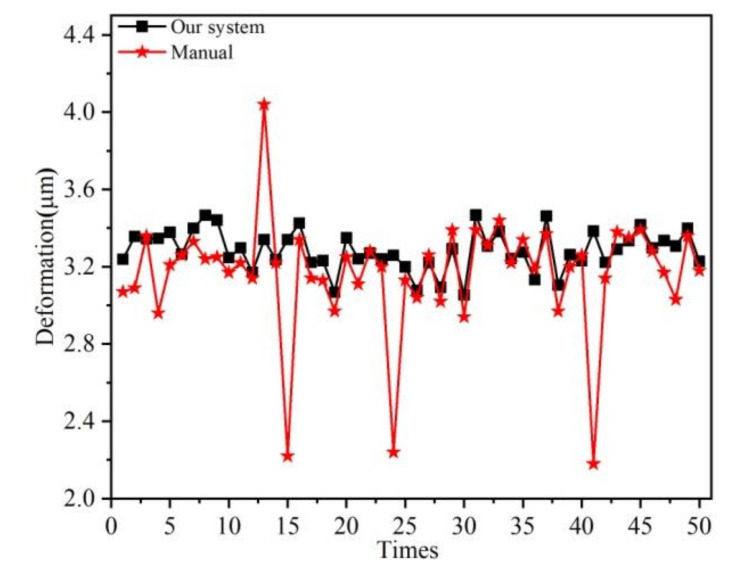
Measurement results of 50 wafers by using our system and the manual method.

Finally, 50 wafers from the same production batch were measured at 100 V by our system and the manual method currently in use, respectively. In principle, they should have close deformations at the same excitation voltage. The measuring results are illustrated in Figure 13, where it is obvious that our system had better reliability.

## 4. Conclusions

A dedicated sorting system for a piezoelectric transducer wafer used in the resonant cavity length adjustment of a ring laser gyro was developed in this work and realized the automatic operation of feeding, measuring, and sorting. It employs a specifically designed operating module, which provides a reliable voltage loading. This ensures the measurement reliability and protects the system hardware and the wafer at the same time. Combined with the dedicated power supply for the piezoelectric transducer and suitable pneumatic inductive micrometers, this system met all the design objectives. The experimental results showed that the repeatability of this system was less than 0.1 µm. Compared with the existing manual measurement, our system possesses better reliability. Compared with the interferometry method, the extreme deviation of measured result was 0.26 µm. It can be concluded that this system provides an effective way to pair piezoelectric transducer wafers used in the resonant cavity length adjustment of a ring laser gyro.

## Figures and Tables

**Table 1:** The key parameters of GTL222-A.

Measurement Range	Maximum Permissible Errors	Hysteresis Error	Repeatability	Measuring Force (Typical Value)
±1.5 mm	0.2 + 2.4 L^2^ μm	0.25 μm	0.015 μm	0.2 N

Note: L is the real stroke of the probe (mm). The measuring force can be adjusted by air pressure control.

**Table 2:** The key parameters of XE501-C.

Basic Output Characteristics	Style
Output voltage V (0~Uo)	300
Average power W/channel	35 ± 20%
Peak power W/channel	100 ± 20%
Average current W/channel	110 ± 20%
Peak current W/channel	330 ± 20%
No-load full bandwidth KHz	10 ± 20%
Voltage stability	<0.1%F.S./8 h
Output ripple mV	5 ± 20%
